# WBC-based segmentation and classification on microscopic images: a minor improvement

**DOI:** 10.12688/f1000research.73315.1

**Published:** 2021-11-17

**Authors:** Xin-Hui Lam, Kok-Why Ng, Yih-Jian Yoong, Seng-Beng Ng

**Affiliations:** 1 Faculty of Computing and Informatics, Multimedia University, Cyberjaya, Selangor, 63100, Malaysia; 2 Faculty of Computer Science and Information Technology, Universiti Putra Malaysia (UPM), UPM Serdang, Selangor, 43400, Malaysia

**Keywords:** Microscopic Images, White Blood Cells, Image Processing, Image Segmentation, Image Classification, Convolutional Neural Network

## Abstract

Introduction

White blood cells (WBCs) are immunity cells which fight against viruses and bacteria in the human body. Microscope images of captured WBCs for processing and analysis are important to interpret the body condition. At present, there is no robust automated method to segment and classify WBCs images with high accuracy. This paper aims to improve on WBCs image segmentation and classification method.

Methods

A triple thresholding method was proposed to segment the WBCs; meanwhile, a convolutional neural network (CNN)-based binary classification model that adopts transfer learning technique was proposed to detect and classify WBCs as a healthy or a malignant. The input dataset of this research work is the Acute Lymphoblastic Leukemia Image Database (ALL-IDB). The process first converts the captured microscope images into HSV format for obtaining the H component. Otsu thresholding is applied to segment the WBC area. A 13 × 13 kernel with two iterations was used to apply morphological opening on image to ameliorate output results. Collected cell masks were used to detect the contour of each cell on the original image. To classify WBCs into a healthy or a malignant category, characteristics and conditions of WBCs are to be examined. A transfer learning technique and pre-trained InceptionV3 model were employed to extract the features from the images for classification.

Results

The proposed WBCs segmentation method yields 90.45% accuracy, 83.81% of the structural similarity index, 76.25% of the dice similarity coefficient, and is computationally efficient. The accuracy of fine-tuned classifier model for training, validation and test sets are 93.27%, 92.31% and 96.15% respectively. The obtained results are high in accuracy and precision are over 96% and with lower loss value.

Discussion

Triple thresholding outperforms K-means clustering in segmenting smaller dataset. Pre-trained InceptionV3 model and transfer learning improve the flexibility and ability of classifier.

## Introduction

According to, ^1^ peripheral blood (PB) or whole blood is the circulating fluid through the entire human body. PB delivers oxygen and nutrients to all the body cells, tissues, and organs, and removes the carbon dioxide and other waste products. It consists of erythrocytes (red blood cells, RBCs), leukocytes (white blood cells, WBCs) and thrombocytes (platelets). RBCs transport oxygen from the lungs to all the body tissues; WBCs fight against the harmful bacteria, parasitic and fungal infections; while platelets clot the blood in wounds on surfaces of the tissue layers. WBCs with a single granulocyte are the monocyte and lymphocyte; while basophil, eosinophil, neutrophil are the WBCs with more than one granulocyte. Lymphocyte can be affected by acute lymphoblastic leukemia (ALL).

Leukemia is a disease formed in tissues that produce large portions of malfunctional and abnormal WBCs that spreads from bone marrow. Based on findings of, ^2^ chronic lymphoblastic leukemia (CLL) (35%) and acute myelogenous leukemia (AML) (32%) are the most common leukemias for adults, while ALL (75%) affects children and teens the most. The World Health Organization (WHO) once stated that ALL is one of the six cancers for children that requires extra attention. ^3^

ALL, AML, CLL and chronic myelogenous leukemia (CML) are leukemia subtypes. Fast-growing cancer in lymphoid cells results in the formation of ALL, ^2^ as opposed to CML; while fast-growing cancer in myeloid cells resulted in the formation of AML, as opposed to CLL. Those over 50 years old and children below 5 years old are the main affected populations of ALL, and the disease can be fatal if not treated earlier. ^4^

PB smear analysis can detect potential disorders and inform health status, while pathology tests can help in tracking the ongoing status of infections, allergies, cancers *etc.* ^5^ A WBC test is one sub-element of a complete blood count (CBC), one of the pathology tests that helps doctors discovers the unexposed infections. The University of Roschester Medical Center (UMRC) has declared that the normal range of WBCs per microliter of blood is 4,000 to 11,000; if WBCs exceed 11,000 per microliter, it is termed as leukocytosis.

Traditional PB smear analysis and CBCs are based on the human inspection. It is laboriously suffered from the intra-observer variability and is not time-efficient or cost efficient. Today, computer-aided diagnosis (CADx) systems are employed and contain four main steps: preprocessing, segmentation, feature extraction, and classification.

Previous work gained 99.14% and 94.12% accuracy for the nucleus segmentation and cell segmentation respectively, while classification accuracy was over 90%. Cell segmentation and binary classification works can be improved further.

### Literature review

Studies by ^6^^,^^7^ summarized WBC segmentation works into pattern recognition-based, deformable model-based, threshold-based, morphological operations-based, and clustering-based segmentation. ^7^ suggested a combination of dual-threshold and morphological operations which achieved 97.85% accuracy. Dual-threshold, binarization, and morphological opening were applied on both preprocessed contrast-stretched grey and H components images. A threshold method was also proposed by other researchers. ^8^^–^^12^ recommended a watershed-based and Otsu threshold-based segmentation which resulted in 99.3% and 93.3% accuracy, respectively.

K-means clustering was another famous segmentation approach. ^13^^–^^16^^,^^20^^,^^32^ applied K-means clustering-based segmentation on the G component of RGB image for two datasets and gained 99.51% and 99.74% accuracy respectively; when applied to a CMYK image, 98.89% accuracy was obtained. ^16^

Deep learning (DL) performed object class prediction by recognizing and learning patterns in visual inputs, making it the state-of-the-art method today. Region of interest (ROI) and neural networks were two other parts of machine learning that were popular nowadays. ^17^ Recommended semantic segmentation that performed whole-slide WBCs segmentation and received 93.34% accuracy. ^18^ Used four-moment statistical features and artificial neural networks (ANN) to segment based on local pixel information, and the overall accuracy was 97%. Work done by ^19^ included WBC localization and Grabcut to perform WBCs segmentation. Edge density (ED) and color contrast (CC) were measured. DL gains high segmentation accuracy; however, it has complex architecture, making it challenging to be a robust and generalized DL model.

For the WBCs classification works, ^20^^–^^22^ employed a deep convolutional neural network (DCNN); ^29^ suggested two methods, neural network (NN) combined with the autoencoders, and a convolutional neural network (CNN); ^23^^–^^25^ also proposed CNN; ^26^^,^^27^ suggested the support vector machine (SVM); ^28^ suggested the combination of K-means neighbours and social spider optimization. Works of ^23^^,^^29^^,^^24^^,^^21^ yielded great outputs. ^24^ used DCNN to classify WBCs into monocytes, neutrophils, eosinophils, and lymphocytes with accuracy of 92.14%, 94.72%, 91.25%, and 94.61% respectively.

This research project is a continuing work that aims to gain improved results for both the WBCs segmentation and classification works. The cell segmentation from the previous work has achieved high accuracy for the nucleus segmentation.

## Method

The input dataset was the ALL-IDB2 dataset provided by the Department of Information Technology at University degli Studi di Milano. ^30^ ALL-IDB2 contains a total of 260 normal and blasts WBCs images collected from blood samples of ALL patients which were designed to test the cell classification efficiency. Classification and labelling process to get the ground truth images was done by the expert oncologists. This dataset was captured using an optical laboratory microscope coupled with a Canon PowerShot G5 camera, saved as JPG format.

### Cell segmentation

**Figure 1.**
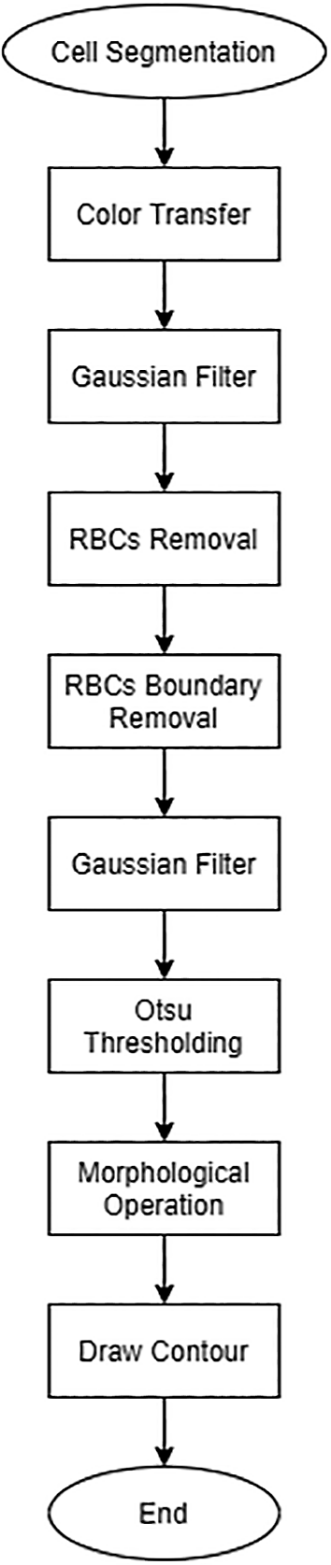
Flowchart of proposed cell segmentation.

**Figure 2.**
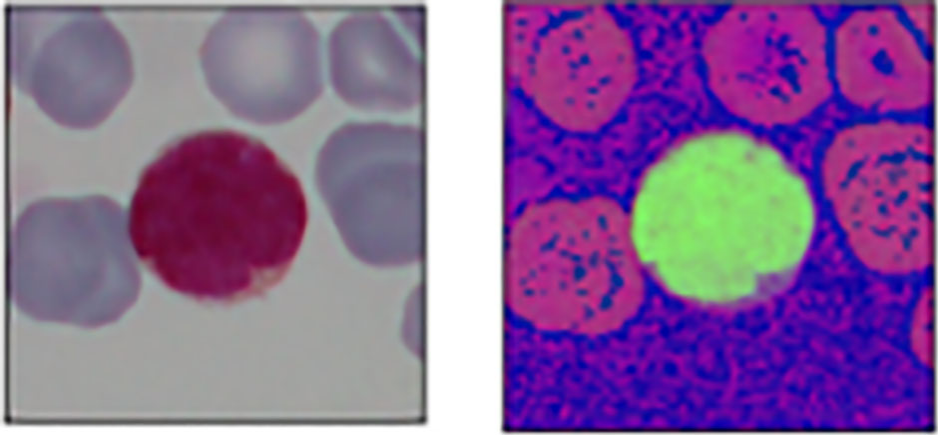
BGR and HSV format input images.

**Figure 3.**
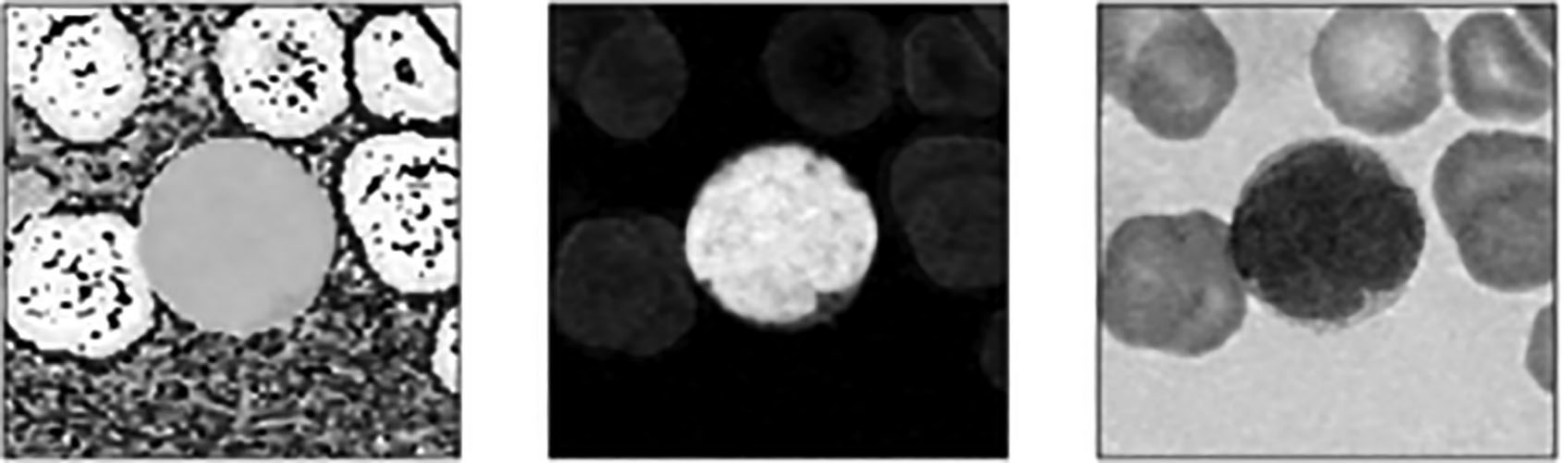
H, S and V channels.

**Figure 4.**
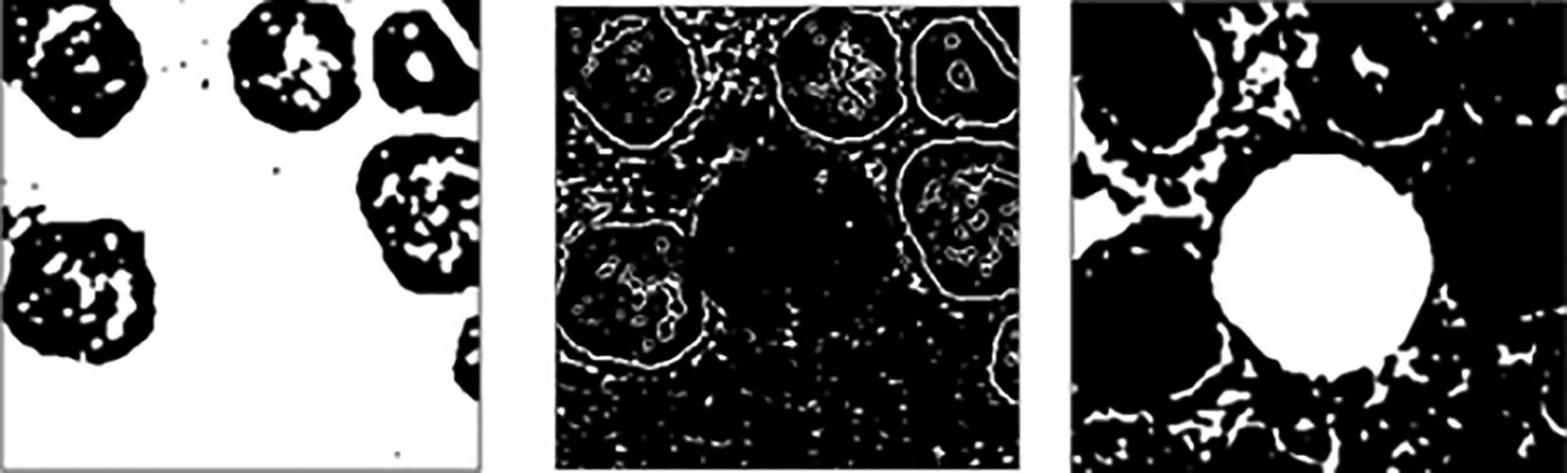
Results obtained from three times of Gaussian filtering and Otsu Thresholding respectively.

**Figure 5.**
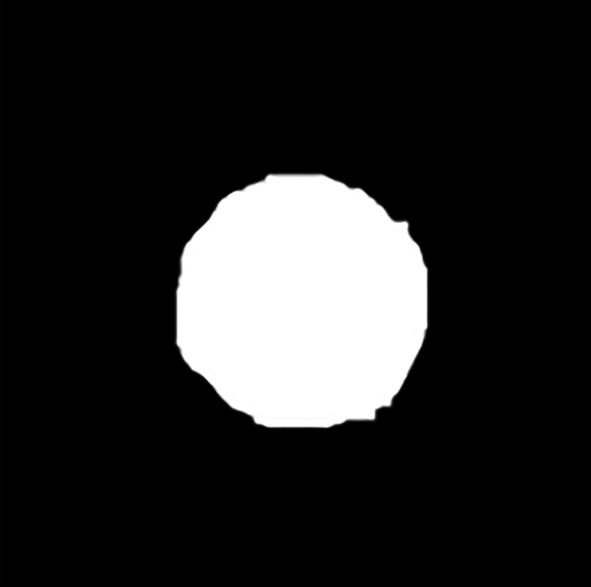
Final segmentation results after applied morphological opening.

**Figure 6.**
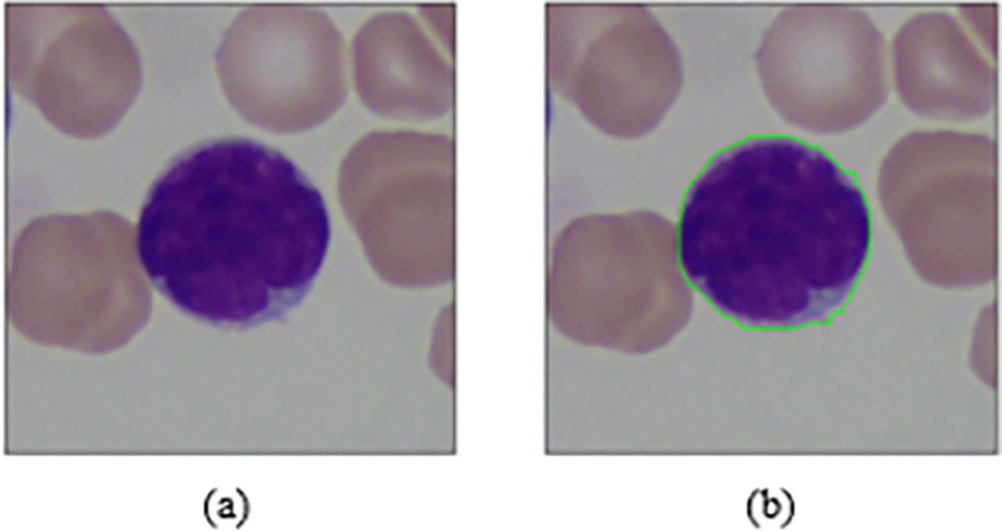
(a) Original RGB image (b) Original RGB image with drawn contour.

Figure 1 shows the overall flowchart of the proposed cell segmentation. H component is extracted from the transformed HSV formatted microscopic image ( Figure 2 and Figure 3). Subsequently, Gaussian filtering and Otsu thresholding ^31^^,^^11^^,^^32^ are applied three times to remove RBCs, RBCs boundaries, and to segment the WBCs. Results for each round can be viewed in Figure 4. Then, a 13 × 13 morphological opening is employed to remove small objects from the foreground to get better results as shown in Figure 5. Finally, cell masks are collected to detect the cell contour from the original RGB formatted image as shown in Figure 6.

### Binary classification

**Figure 7.**
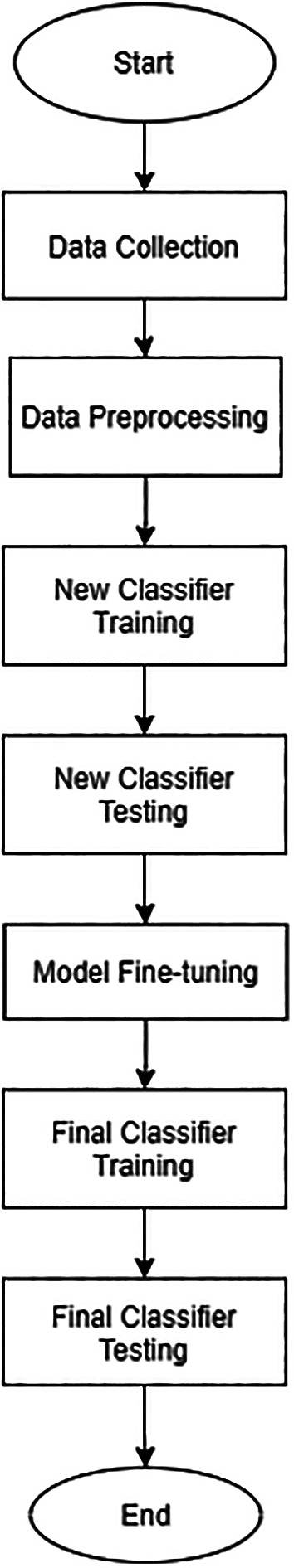
Flowchart of building up proposed binary CNN classifier model.

Figure 7 shows the flowchart of the formation of the proposed binary WBCs classifier model. Characteristics and conditions of WBCs are crucial for this work.

A pre-trained InceptionV3 model is employed. ^33^^–^^35^ It is used to extract features in a data preprocessing step which was inspired by. ^12^ Also, the transfer learning technique is adopted. Dataset is divided into training, validation and testing sets, 208 images for training set, while validation and test sets each contains 26 images. All the input images are re-sized to 150 × 150 pixels and are normalized to (−1, 1) to fit into InceptionV3 model.

**Figure 8.**
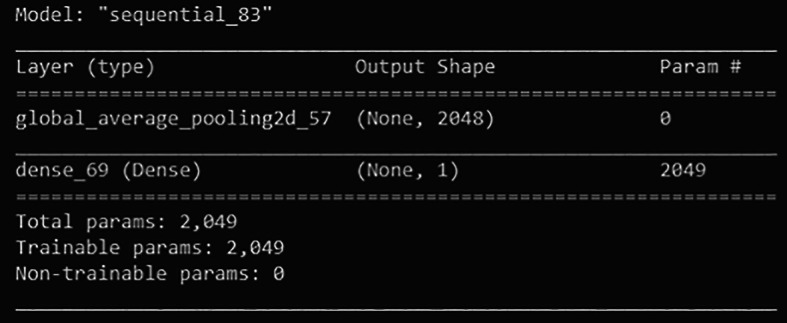
Architecture of the newly defined classifier.

**Figure 9.**
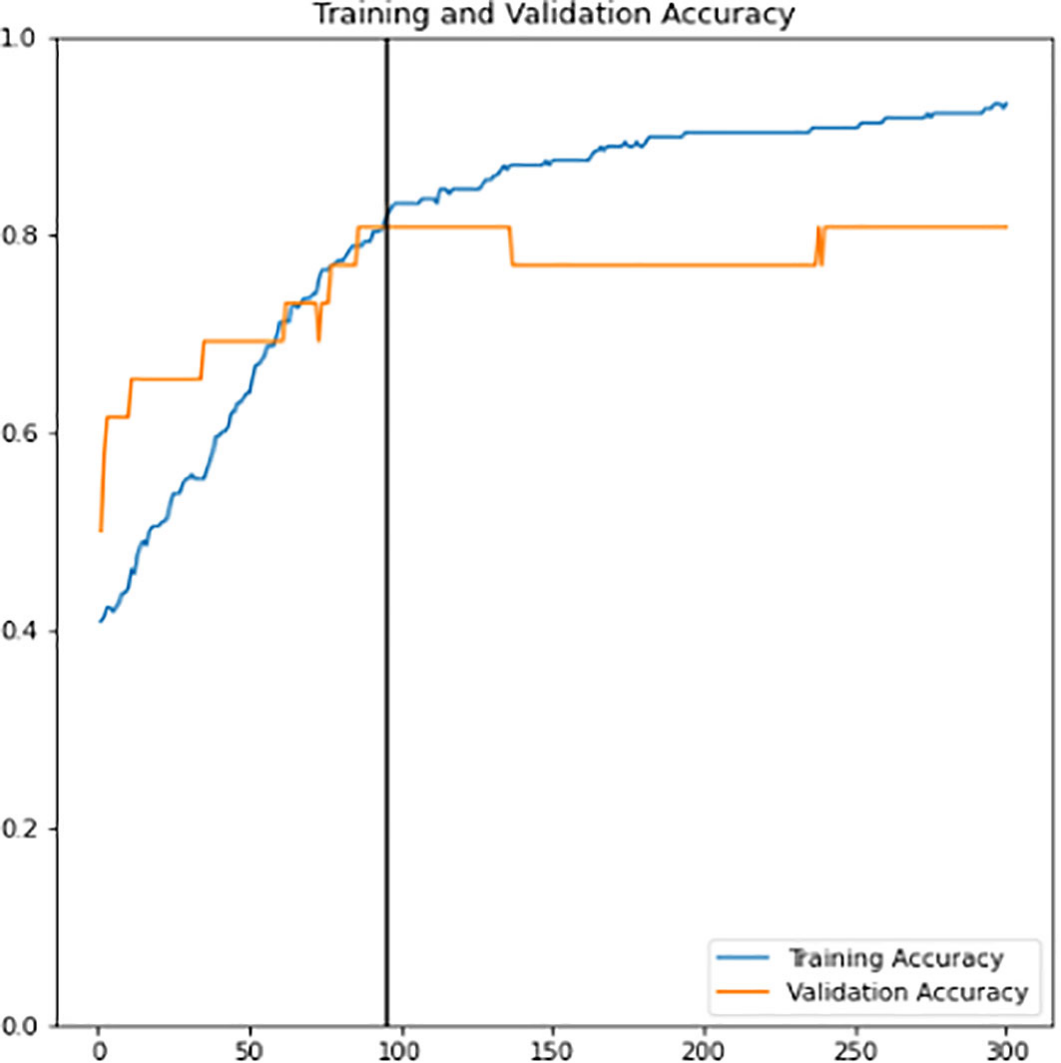
Training learning curve of accuracy for training and validation sets of newly defined classifier.

**Figure 10.**
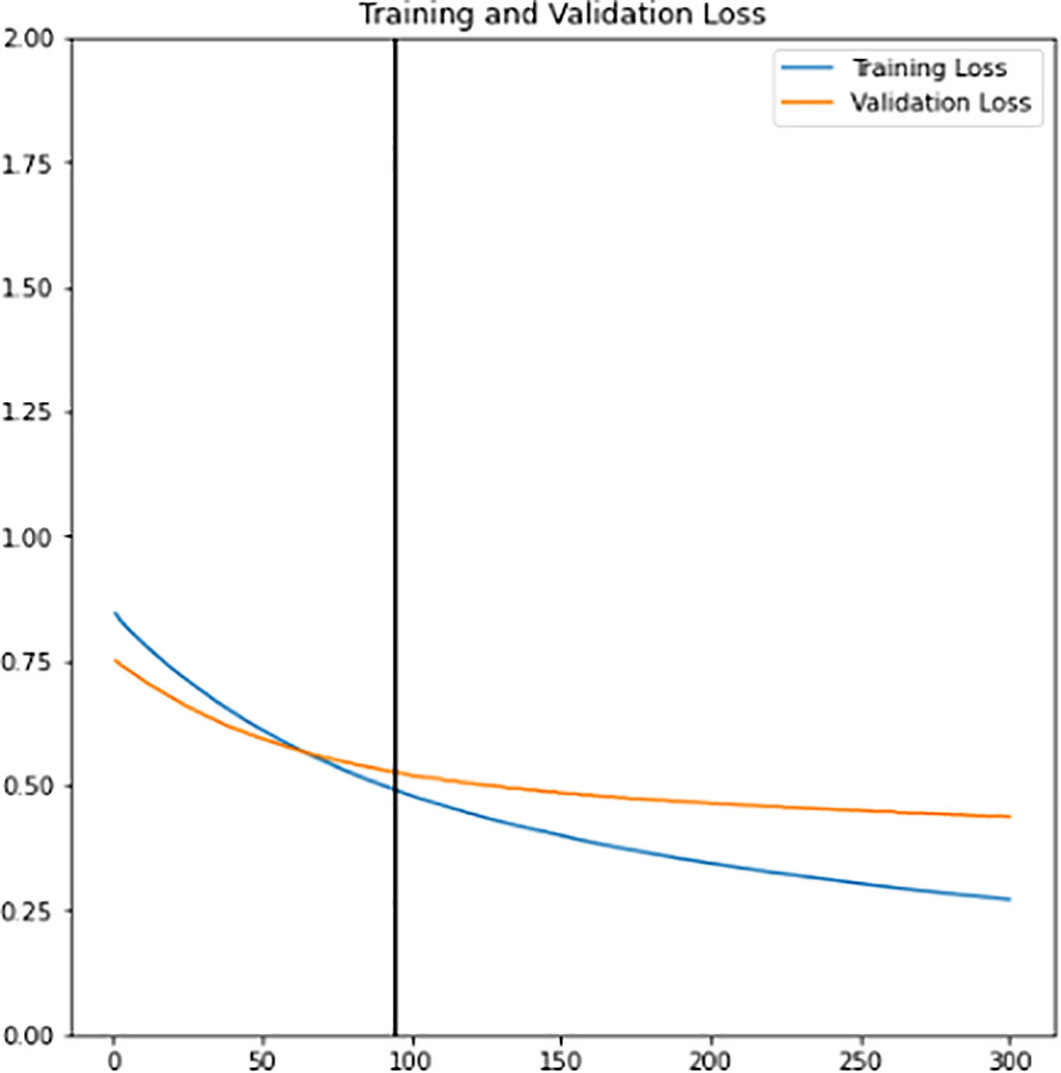
Training learning curve of loss for training and validation sets of newly defined classifier.

**Figure 11.**
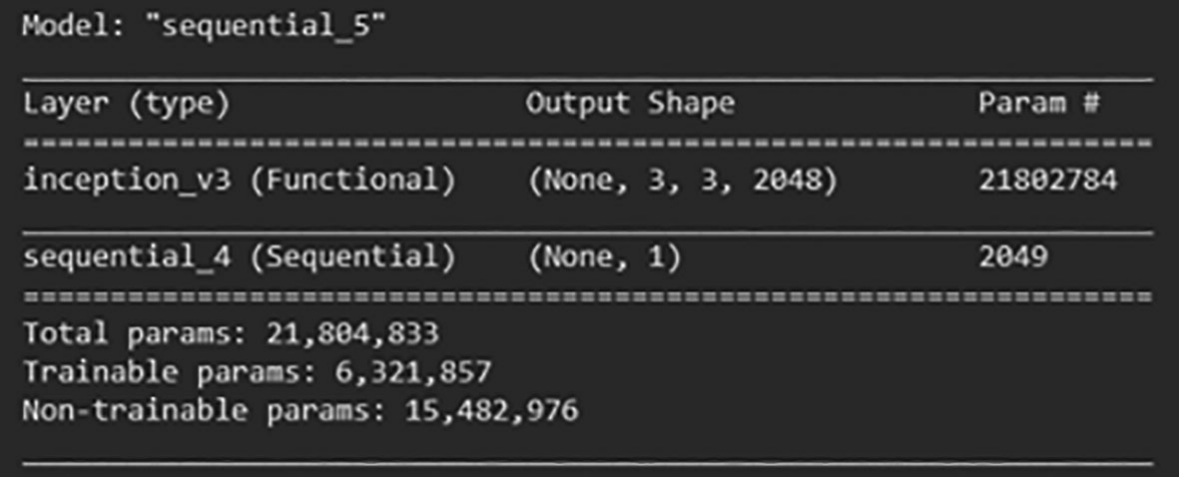
Architecture of fine-tuned classifier.

First, a new classifier is built and its architecture is shown in Figure 8. It has 2049 trainable parameters, global average pooling that forms a feature map to prevent overfitting, and a dense layer that applies sigmoid activation to do the binary classification. The pre-trained InceptionV3 model extracts all the features from the training and validation sets. Next, the model is trained for 300 epochs. The training learning curves graph (accuracy and loss) of the newly defined classifier are shown in Figure 9 and Figure 10. The point of interception has the highest validation accuracy and smaller differences between training and validation accuracy. Hence, the epoch value along with the lead of intersection between the two curve lines is the best epoch chosen as a final classifier. New classifier will then be combined with the pre-trained InceptionV3 to form the final classifier. As shown in Figure 11, out of 21,804,833 parameters, the final classifier had a total of 6,321,857 trainable parameters.

**Figure 12.**
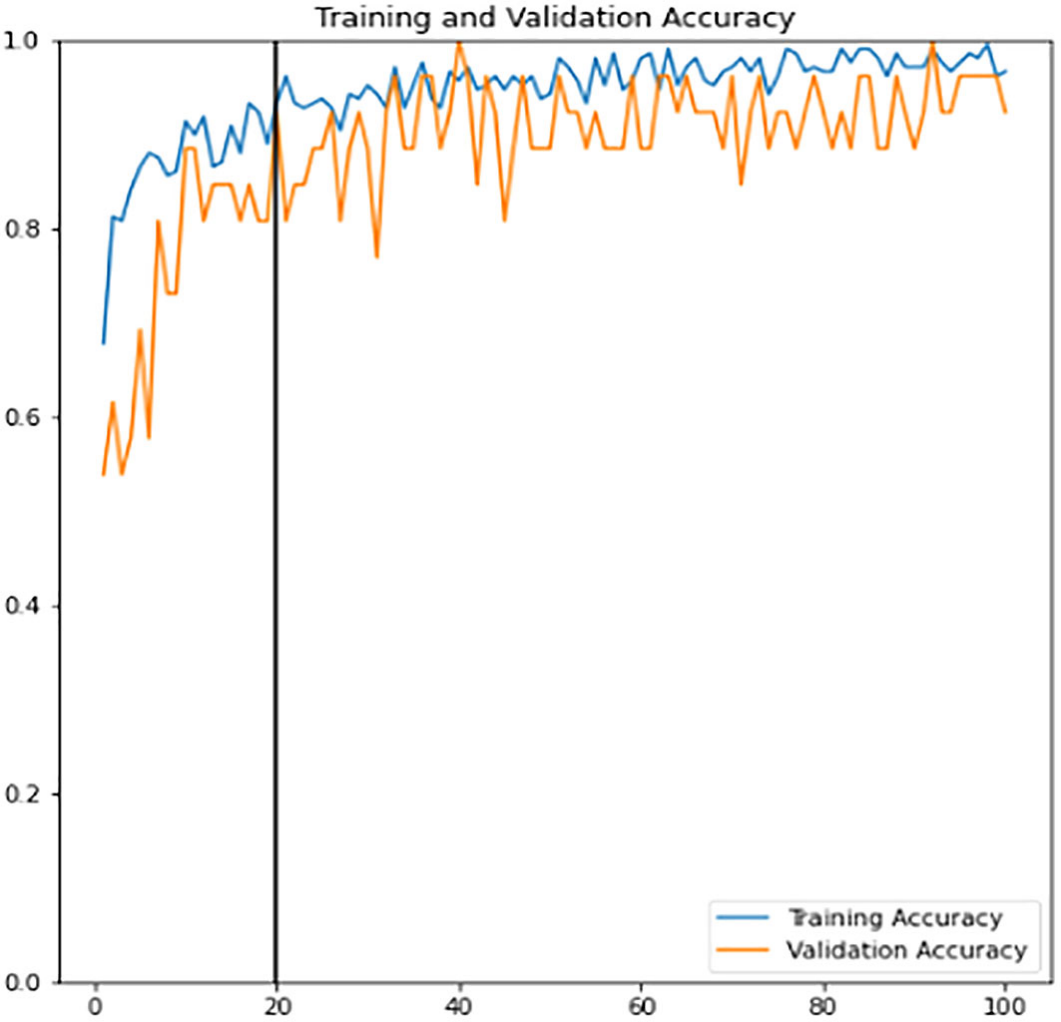
Training learning curve of accuracy for training and validation sets of the fine-tuned classifier.

**Figure 13.**
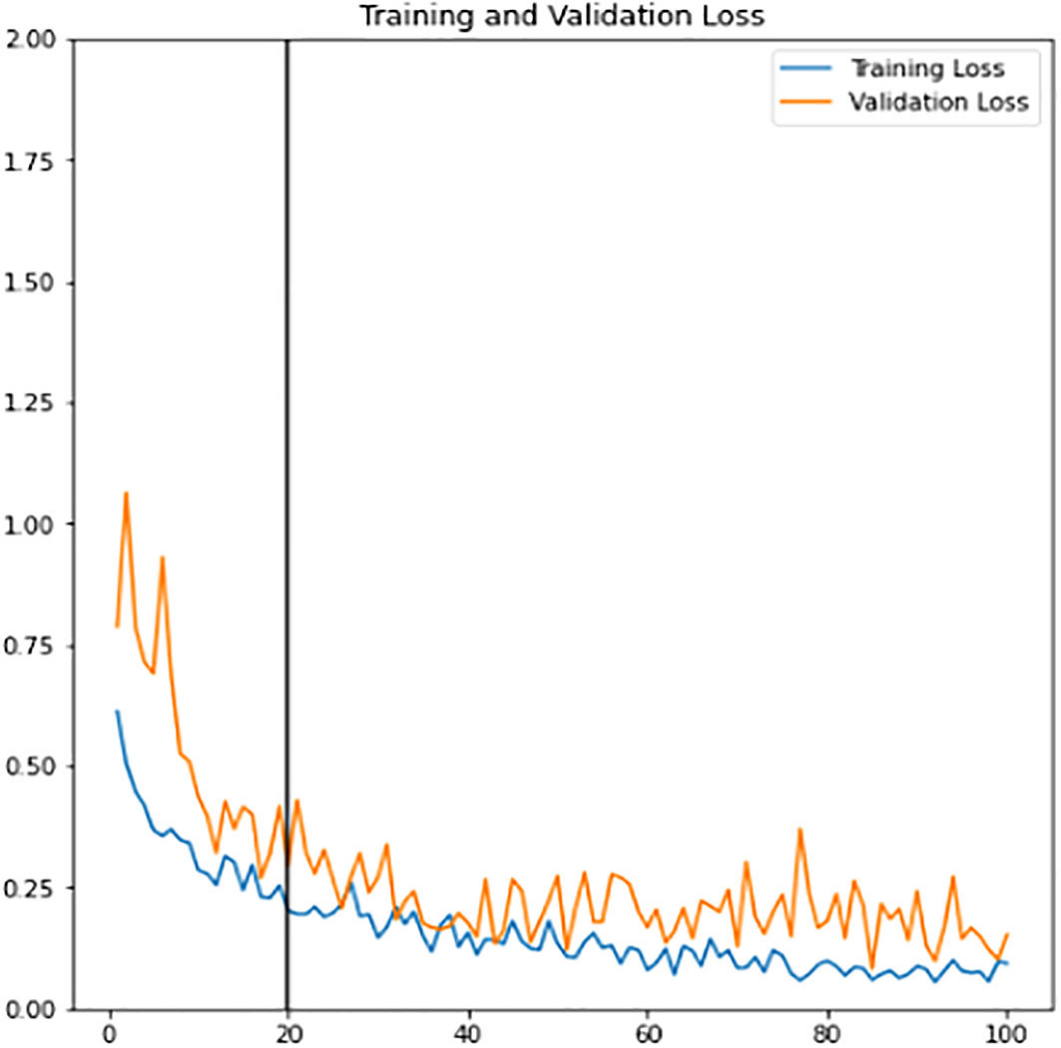
Training learning curve of loss for training and validation sets of the fine-tuned classifier.

Data augmentation increases the diversity of data using various techniques such as flipping, rotation, translation *etc.* The benefit that comes along with this technique is reducing the model bias tendency towards a particular class of data. Thus, it is applied to both the training and validation sets to allow the model to learn better and to reduce the overfitting consequences. The training learning curves graph (accuracy and loss) of the fine-tuned classifier model can be viewed in Figure 12 and Figure 13. Epoch with the highest validation accuracy and lowest validation loss is chosen as the final classifier.

## Results

### Cell segmentation

**Figure 14.**
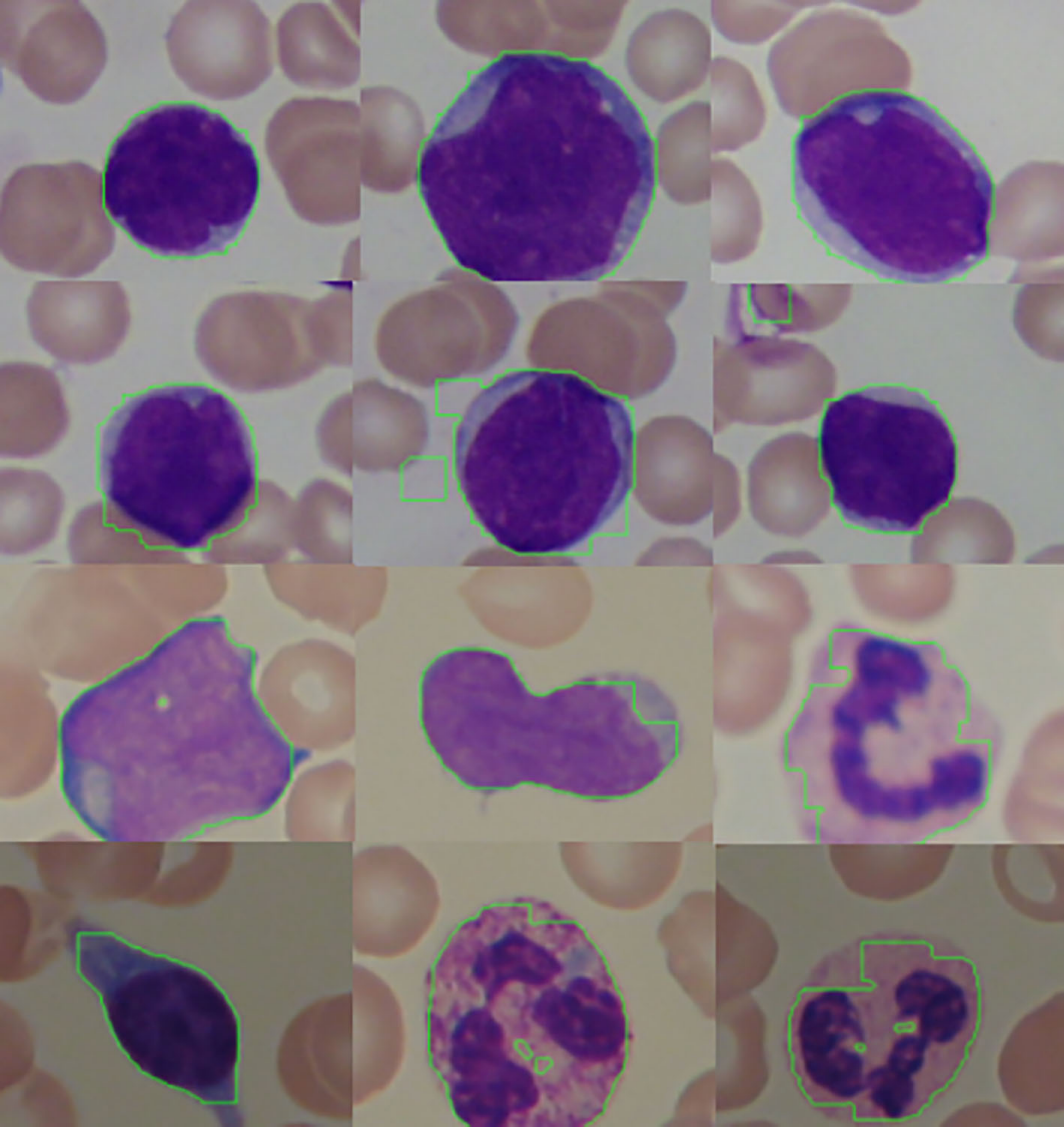
Results of proposed cell segmentation.

Figure 14 shows some results of the proposed cell segmentation that attained 90.45%, 93.91%, 76.25% and 0.0186 seconds for the average accuracy, structural similarity, dice similarity coefficient and computational time respectively. The final results achieve high accuracy and similarity, over the average overlapping between the ground truth and the original images with short computation time. In short, the proposed cell segmentation method makes a performance.

The sample results shown in Figure 14 demostrate that the first row yields an excellent segmentation outcomes while the following rows of images weren’t. This may be due to the undesirable illuminations and blur levels. In addition, the inconspicuous difference between the foreground (WBC) and the background caused by the uneven acquisition can be another factor that affects the final results.

**Figure 15.**
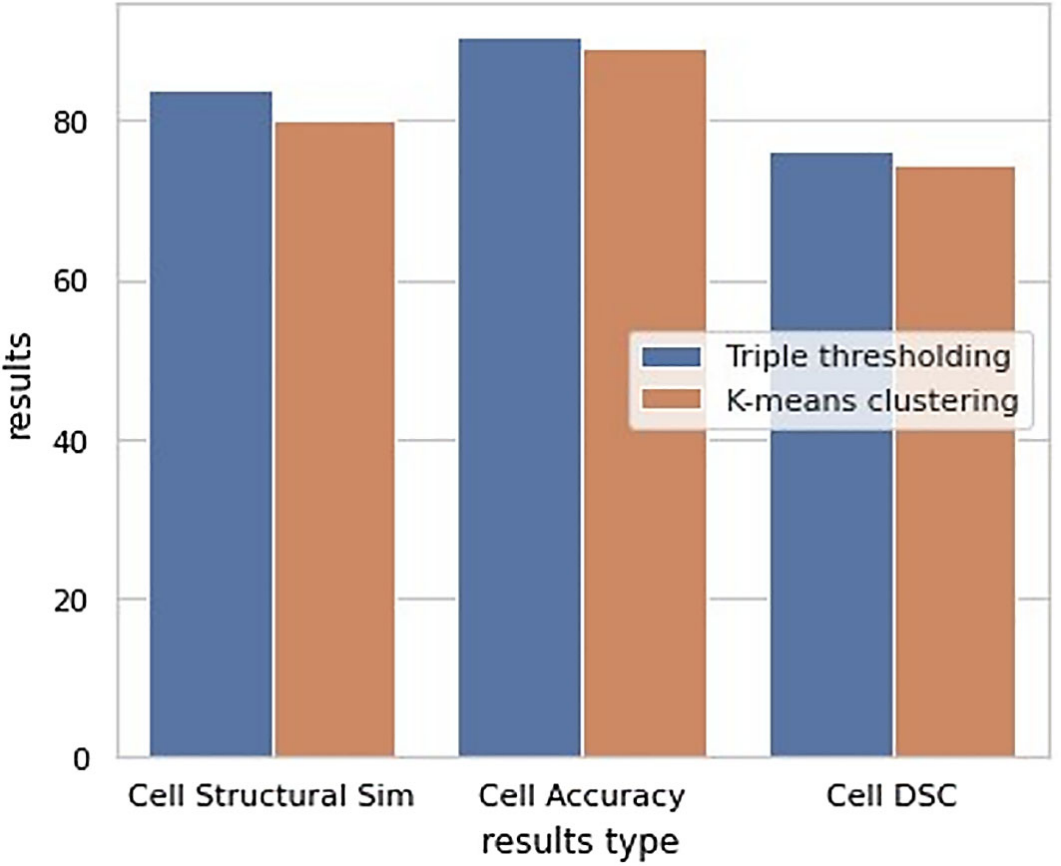
Grouped bar chart for comparison between the K-means clustering and the proposed triple thresholding method.

Table 1 and the bar chart in Figure 15 show the comparison between the K-means clustering and the proposed triple thresholding method. This work obtained higher accuracy in a shorter time.

**Table 1.** Comparison between previous work (K-means clustering) and proposed work (Triple thresholding).

	K-means clustering	Triple thresholding
Accuracy	0.8905	0.9045
Structural similarity	0.8005	0.8381
Dice similarity coefficient	0.7438	0.7625
Computational time	1.1935	0.0186

### Binary classification

Table 2 shows the performance of the newly defined classifier; while Table 3 shows the performance of the fine-tuned classifier.

**Table 2.** Performance of newly defined classifier.

	Training set	Validation set	Test set
Accuracy	0.8173	0.8077	0.8974
Loss	0.4906	0.5274	0.4982
Precision	0.8000	0.7857	0.9444
Recall	0.8462	0.8462	0.8308
True positive	88.0000	11.0000	8.6667
True negative	82.0000	10.0000	10.6667
False positive	22.0000	3.0000	0.6667
False negative	16.0000	2.0000	1.6667

**Table 3.** Performance of fine-tuned classifier.

	Training set	Validation set	Test set
Accuracy	0.9327	0.9231	0.9615
Loss	0.2028	0.2941	0.2229
Precision	0.9412	0.9231	1.0000
Recall	0.9231	0.9231	0.9231
True positive	96.0000	12.0000	12.0000
True negative	98.0000	12.0000	13.0000
False positive	6.0000	1.0000	0.0000
False negative	8.0000	1.0000	1.0000

It is observed that the accuracy, precision, recall, true positive and true negative for all the three sets of data increased while loss, false positive and false negative results decreased after the fine-tuned stage. This indicates that the fine-tuned classifier is a robust and good fitted classifier that can accurately predict and make fewer errors.

**Figure 16.**
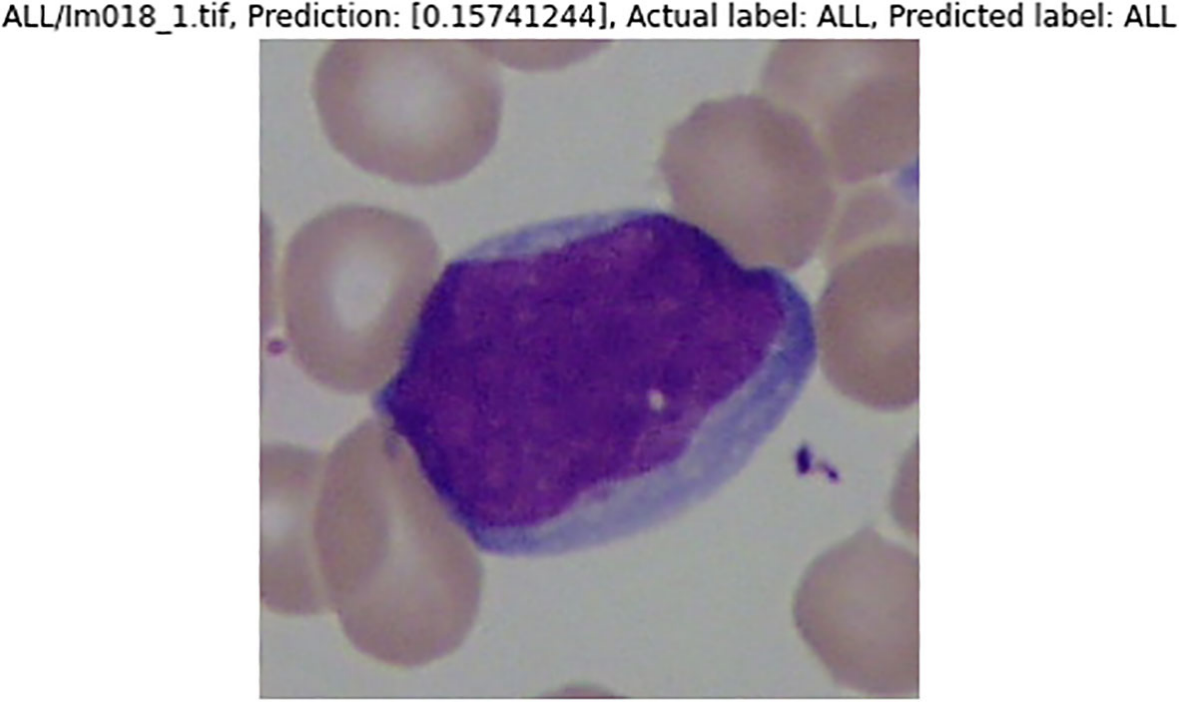
Sample ALL WBCs image being tested.

**Figure 17.**
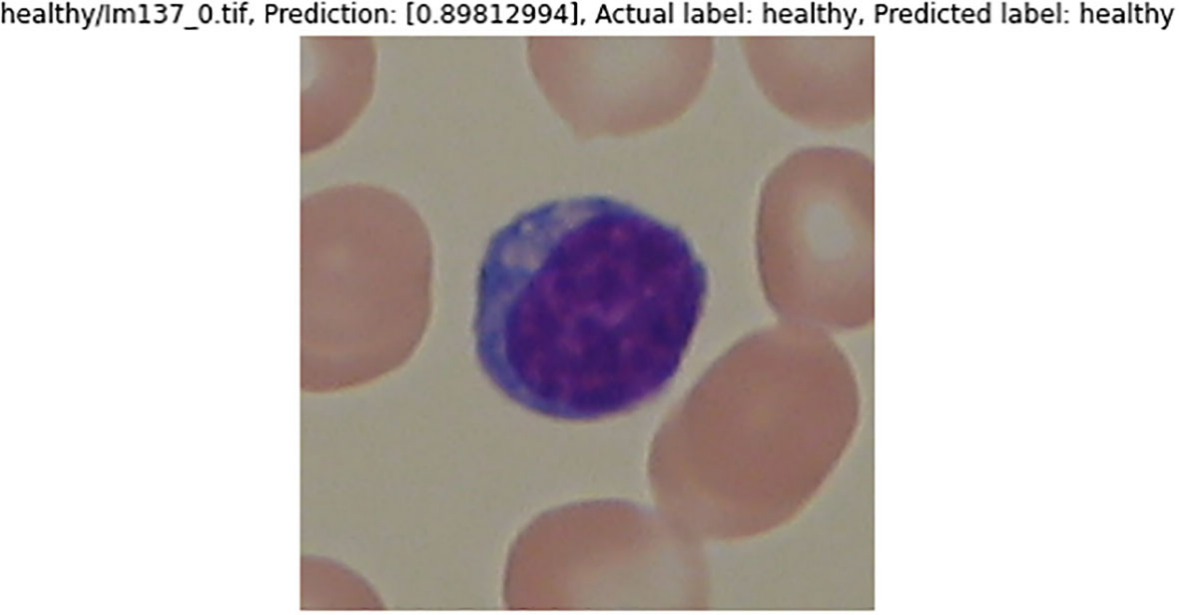
Sample healthy WBCs image being tested.

Figure 16 and Figure 17 are the samples of the binary classification results. If the prediction value is over 0.5, they are considered as healthy WBCs; otherwise, they are ALL WBCs. Results of the predicted label by the classifier and the actual label are the same. Both the sample images are classified correctly.

**Figure 18.**
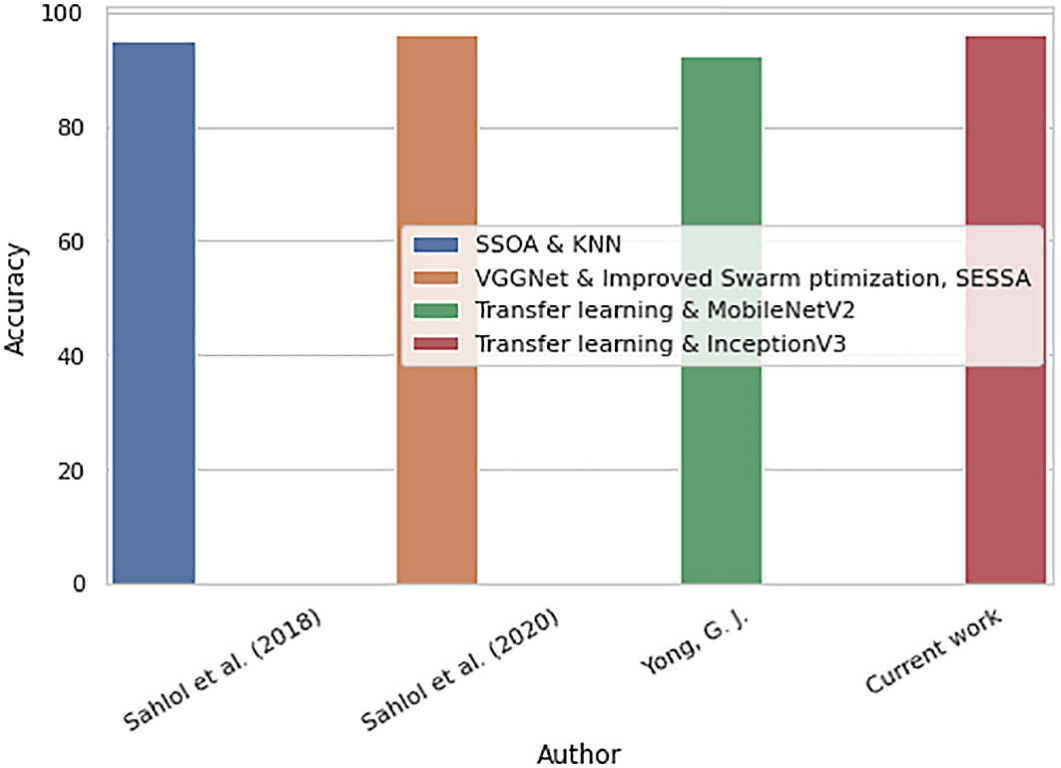
Bar chart for comparisons between previous works and proposed work.

Comparisons between the previous works and the proposed work for the binary classification are shown in Table 4 and the bar chart in Figure 18. The proposed binary classification work outperforms previous works with the highest accuracy, 96.15%. The proposed work is able to classify the WBCs into healthy and malignant groups accurately.

**Table 4.** Comparisons between previous works and proposed work.

	Proposed method	Accuracy (%)
[ 29]	Feature extraction using SSOA and KNN	95.23
[ 36]	VGGNet features extractor and Improved Swarm optimization (SESSA) features selector	96.11
[ 12]	Transfer learning on MobileNetV2 classifier	92.31
**Proposed work**	**Transfer learning on pre-trained InceptionV3 CNN classifier**	**96.15**

## Discussion

The proposed method in this study shows a new possible approach or direction for the future research work on WBC cell segmentation and binary classification.

In the comparison table between the previous works and the proposed work for the cell segmentation, it is proven that the threshold-based segmentation can outperform the K-means clustering when dealing with a small dataset. Otsu thresholding uses the global thresholding while the K-means clustering uses the local thresholding to perform the segmentation. Otsu thresholding calculates the optimum thresholding after distributing the pixels into foreground and background classes. K-means clustering determines the k centroids, evaluates pixels and groups the similar pixels into the same cluster. Most images of the ALL-IDB2 contain large foreground (WBC) size leading to a good bimodal distribution of the histogram where Otsu thresholding is more capable and expertise than K-means clustering to perform segmentation due to the thresholding technique used. The pre-processing and post-processing are crucial as they can very much affect the final output of the Otsu thresholding.

On the other hand, the proposed binary WBC classification that uses the pre-trained InceptionV3 and transfer learning technique yields 96% of accuracy. The pre-trained classifier model is trained with a large dataset, hence, it eliminates the need to build a classifier model from scratch. This makes it able to extract features better than VGGNet or SSOA in previous works. Transfer learning technique reuses pre-trained model on a different but similar problem is a noble approach. This gives the model a higher learning rate during the training. It is not only more intelligent but also able to accomplish works in higher performance. Using pre-trained and transfer learning approaches, the binary classifier model is more brilliant and flexible than the previous research. It can adapt quickly and use the best-fitted methods to perform classification on the dataset. Also, the InceptionV3 model requires fewer generated parameters of networks as compared with VGGNet. Hence, the final results are better than the previous works when combined with the binary WBCs classification. From Table 4, it is observed that the proposed work has a potential to be considered for the future application.

## Conclusions

This research focuses on improving the WBC cell segmentation and binary classification works. A public dataset, ALL-IDB2 is used to perform the cell segmentation and binary classification. Triple thresholding method is proposed to achieve the first objective of this research and the results are 90.45%, 83.81%, 76.25%, and 0.0186 seconds for the accuracy, structural similarity index, dice similarity coefficient, and computation time respectively. Combining the pre-trained InceptionV3 model and the transfer learning technique produce over 96% accuracy and the precision with a lower loss value was suggested to accomplish the second objective of this research. The overall performance for both the segmentation and classification works have been improved.

## Author contributions

LamXH, NgKW, YoongYJ and NgSB conceived the presented idea. LamXH carried out the experiment and wrote the manuscript. NgKW, YoongYJ and NgSB supervised the project and provided feedback.

## Ethics

Ethical Approval Body: Research Ethic Committee 2021, Multimedia University

Ethical Approval Number: EA1552021

## Data availability

Data were obtained from Acute Lymphoblastic Leukemia Image Database for Image Processing (ALL-IDB) ( https://homes.di.unimi.it/scotti/all/).

This dataset was not generated nor is it owned by the authors of this article; the listed owners is the Department of Computer Science - Università degli Studi di Milano. Therefore, neither the authors nor F1000Research are responsible for the content of this dataset and cannot provide information about data collection.
