# Supplementary material for: WBC-based segmentation and classification on microscopic images: a minor improvement
Source: F1000Res. 2021 Nov 17;10:1168. [Version 1] doi: 10.12688/f1000research.73315.1 (PMC8976187; doi:10.12688/f1000research.73315.1)
Supplement: Peer review history

# Peer review: Reviewer response for version 1

**DOI:** 10.5256/f1000research.76958.r120327

**Published:** 2022-04-01

**Authors:** Chiung Ching Ho

**Affiliations:** 1 Department of Computing and Information Systems, Sunway University, Subang Jaya, Malaysia

This paper reports on an effort to segment and classify microscopic images.

1. I wonder how the authors define "a minor improvement"? The baseline results on the original dataset should be listed, if applicable.
2. Perhaps authors should consider if the listed publications at https://homes.di.unimi.it/scotti/all/ are necessary to be cited.
3. Why was InceptionV3 selected and not any other pre-trained model?
4. Can the authors comment on the work entitled "IoT Application of Transfer Learning in Hybrid Artificial Intelligence Systems for Acute Lymphoblastic Leukemia Classification" ^1^? It uses low power IoT devices with an encoder and a classification head. And achieves higher accuracy - what would be a fair comparison between this work and the author's work?

Is the work clearly and accurately presented and does it cite the current literature?

Partly

If applicable, is the statistical analysis and its interpretation appropriate?

Yes

Are all the source data underlying the results available to ensure full reproducibility?

Yes

Is the study design appropriate and is the work technically sound?

Yes

Are the conclusions drawn adequately supported by the results?

Yes

Are sufficient details of methods and analysis provided to allow replication by others?

Yes

Reviewer Expertise:

Deep Learning, Machine Learning

I confirm that I have read this submission and believe that I have an appropriate level of expertise to confirm that it is of an acceptable scientific standard.

## References

[1] : IoT Application of Transfer Learning in Hybrid Artificial Intelligence Systems for Acute Lymphoblastic Leukemia Classification. *Sensors (Basel)* .2021;21(23) : 10.3390/s21238025 10.3390/s21238025 34884029PMC8659925

# Peer review: Reviewer response for version 1

**DOI:** 10.5256/f1000research.76958.r100625

**Published:** 2021-12-23

**Authors:** Naresh Babu Muppalaneni

**Affiliations:** 1 Department of Computer Science and Engineering, National Institute of Technology, Silchar, Assam, India

The authors have used a novel methodology for White Blood Cell (WBC) segmentation and classification. They have proposed a new classifier and fine-tune the classifier for better accuracy. The results are prominent as the accuracy is high compared to the existing methods in Hedge *et al. (*2019) ^1^, Prellberg et al. (2019) ^2^ and Sahlol *et al. *(2020) (references 29, 36, and 12 respectively in the article). The results are tabulated and represented in the form of graphs and charts.

 The authors have used Gaussian filtering with Otsu thresholding for three times with a 13 x 13 filter for segmentation of WBC and to remove Red Blood Cells (RBC). What is the significance of the 13 x 13 filter? The reason is not found in the paper.

 The authors experimented with neural networks combined with autoencoders for improved accuracy. The authors have used various pre-trained models for classification and also performed comparative studies with the existing techniques from the literature.

Is the work clearly and accurately presented and does it cite the current literature?

Yes

If applicable, is the statistical analysis and its interpretation appropriate?

Yes

Are all the source data underlying the results available to ensure full reproducibility?

Yes

Is the study design appropriate and is the work technically sound?

Yes

Are the conclusions drawn adequately supported by the results?

Yes

Are sufficient details of methods and analysis provided to allow replication by others?

Yes

Reviewer Expertise:

Myself working in the area of machine learning, deep learning and made good number of publications in bioinformatics, machine learning.

I confirm that I have read this submission and believe that I have an appropriate level of expertise to confirm that it is of an acceptable scientific standard.

## References

[1] : Comparison of traditional image processing and deep learning approaches for classification of white blood cells in peripheral blood smear images. *Biocybernetics and Biomedical Engineering* .2019;39(2) : 10.1016/j.bbe.2019.01.005 382-392 10.1016/j.bbe.2019.01.005

[2] : Acute Lymphoblastic Leukemia Classification from Microscopic Images Using Convolutional Neural Networks. 10.1007/978-981-15-0798-4_6 53-61 10.1007/978-981-15-0798-4_6
